# Natural variation of H3K27me3 distribution between two *Arabidopsis *accessions and its association with flanking transposable elements

**DOI:** 10.1186/gb-2012-13-12-r117

**Published:** 2012-12-19

**Authors:** Xue Dong, Julia Reimer, Ulrike Göbel, Julia Engelhorn, Fei He, Heiko Schoof, Franziska Turck

**Affiliations:** 1 Max Planck Institute for Plant Breeding Research, Carl von Linne Weg 10, 50829 Köln, Germany; 2 Current address: Institute for Evolution and Biodiversity, University of Münster, Hüfferstr. 1, 48149 Münster, Germany; 3 Current address: University of Bonn, Institute of Crop Science and Resource Conservation, Crop Bioinformatics, Katzenburgweg 2, 53115 Bonn, Germany

## Abstract

**Background:**

Histone H3 lysine 27 tri-methylation and lysine 9 di-methylation are independent repressive chromatin modifications in *Arabidopsis thaliana*. H3K27me3 is established and maintained by Polycomb repressive complexes whereas H3K9me2 is catalyzed by SUVH histone methyltransferases. Both modifications can spread to flanking regions after initialization and were shown to be mutually exclusive in *Arabidopsis*.

**Results:**

We analyzed the extent of natural variation of H3K27me3 in the two accessions Landsberg *erecta *(L*er*) and Columbia (Col) and their F1 hybrids. The majority of H3K27me3 target genes in Col were unchanged in L*er *and F1 hybrids. A small number of L*er*-specific targets were detected and confirmed. Consistent with a *cis*-regulatory mechanism for establishing H3K27me3, differential targets showed allele-specific H3K27me3 in hybrids. Five L*er*-specific targets showed the active mark H3K4me3 in Col and for this group, differential H3K27me3 enrichment accorded to expression variation. On the other hand, the majority of L*er*-specific targets were not expressed in Col, L*er *or 17 other accessions. Instead of H3K27me3, the antagonistic mark H3K9me2 and other heterochromatic features were observed at these loci in Col. These loci were frequently flanked by transposable elements, which were often missing in the L*er *genome assembly.

**Conclusion:**

There is little variation in H3K27me3 occupancy within the species, although H3K27me3 targets were previously shown as overrepresented among differentially expressed genes. The existing variation in H3K27me3 seems mostly explained by flanking polymorphic transposable elements. These could nucleate heterochromatin, which then spreads into neighboring H3K27me3 genes, thus converting them to H3K9me2 targets.

## Background

Eukaryotic nuclear DNA is organized in a higher order chromatin structure that enables the required dense packaging, but also limits access to nuclear DNA. To overcome the chromatin barrier, chromatin associated complexes enable local and temporal opening of chromatin so that crucial processes such as transcription, replication and DNA repair can take place. In contrast, mechanisms that reinforce chromatin compaction have been integrated in pathways that confer stable gene repression. In particular, two evolutionarily conserved pathways of chromatin-mediated gene repression have been described [1-3]. The SU(VAR)3-9 pathway and the related SUVH pathway in plants effectively silence target loci due to heterochromatin formation [4,5]. In plants, gene silencing is in general stably inherited throughout meiosis [6]. Main targets of the SUVH pathway are transposable elements (TEs) and derived repeats. In contrast, the Polycomb group (PcG) pathway causes persistent but reversible repression of euchromatic genes that can be inherited throughout mitosis but is reset during meiosis. The PcG pathway plays a crucial role in the regulation of genes that have important developmental functions [7].

Central to both pathways are SET domain histone methyltransferases (HMTs) that specifically modify lysine residues at either position 9 or 27 of histone H3, both sites being embedded in evolutionarily conserved ARKS motifs in the amino-terminal tail of H3 [8]. Histone tails and their modifications do not contribute to nucleosome structure or stability, but serve as recognition sites for chromatin-associated complexes that are required for chromatin compaction and gene repression [9]. For both repressive pathways, a mechanism designated as 'spreading' has been described. In the spreading model, the HMT complexes are first recruited to a nucleation region by a specific signal, which could be a DNA-binding protein or non-coding RNA. The histone modification then spreads from this nucleation site to neighboring regions due to an auto-catalytic process [10]. The amplification is dependent on the direct or indirect recruitment of the HMT complex by its own target modification. Such an auto-catalytic loop has been described for mammalian SU(VAR)3-9, which is known to interact physically with HETEROCHROMATIN PROTEIN 1 (HP1) [4]. HP1 directly binds the H3K9me2 and H3K9me3 modification through its chromodomain [4]. In the PcG pathway, it was shown that an accessory component of the HMT containing complex interacts with the H3K27me3 mark [11,12].

In *Arabidopsis*, the production of small interfering RNAs (siRNAs) of 23 to 24 nucleotides in length leads to the recruitment of several HMTs of the SUVH-type to complementary sites, resulting in di-methylation of lysine 9 of H3 [13]. These siRNAs are generated from double-stranded RNA transcripts and primarily target TEs or related repetitive DNA. The chromo-domain containing DNA-methylase Chromo-methylase 3 (CMT3) is recruited by H3K9me2 and this contributes to an increase in cytosine DNA-methylation at the sites marked by H3K9me2 [14]. However, DNA-methylation can also be recruited to target sites by siRNAs independently of H3K9me2 and may in fact result in increased recruitment of H3K9me2, both mechanisms contributing to the spreading of H3K9me2 domains in plants [15]. In animals, boundary sequences that carry H2Bub1 have been shown to limit spreading of heterochromatin into neighboring regions [16].

The SET domain component of Polycomb repressive complex (PRC)2 tri-methylates lysine 27 of H3 and this modification is recognized by PRC1. In contrast to H3K9me2, the H3K27me3 modification is not correlated with cytosine methylation in plants [17,18]. In plants, the chromodomain protein LIKE HETEROCHROMATIN PROTEIN 1 (LHP1) recognizes the H3K27me3 modification as part of a PRC1-like complex, although its closest animal homologs, the HP 1 family members, are an integral part of the SU(VAR)3-9 pathway. In plants and animals, PRC1 also comprises RING domain proteins that ubiquitinate lysine residues within the body of H2A [19,20]. In animal models, it has been shown that H2A ubiquitination is crucial for chromatin compaction and target gene repression [21]. Different modes of PRC2 recruitment to target sites have been reported and it is possible that several mechanisms act synergistically. In fruitflies, Polycomb response elements are regulatory regions that contain arrays of *cis*-elements involved in PRC2 recruitment [22]. In addition, intergenic non-coding RNAs such as the *HOTAIR *transcript have been implicated in PRC2 targeting [23]. Recently, the intron-encoded non-coding RNA *COLDAIR *was shown to specifically bind the plant PRC2 complex to stably repress a target gene [24].

Several genomic studies in *Arabidopsis thaliana *identified a large number of genes (about 15% of all genes in the annotated genome of the Columbia (Col) accession) as marked with H3K27me3 [25-27]. H3K27me3-enriched regions often span large proportions of their target gene, but they rarely spread into neighboring annotated units [25,26]. Most H3K27me3-marked genes show low expression levels throughout development or are expressed in a highly tissue-specific manner [26].

Here, we address the question of to what extent H3K27me3 distribution varies between two accessions of *Arabidopsis *and point out genomic features that may explain this variation at a genetic and epigenetic level. We discovered that H3K27me3 targets specific to one accession are often repressed by other repressive marks such as H3K9me2 and DNA methylation in the reference accession. The insertion of TEs in the vicinity of accession-specific H3K27me3 targets followed by heterochromatin spreading and invasion of H3K27me3 domains seems to play a major role in creating the epigenetic variation. We could show that variation in H3K27me3 enrichment was inherited strictly in *cis *in F1 hybrids between both accessions.

## Results

### Col and L*er *have highly similar H3K27me3 profiles

**Figure 1:**
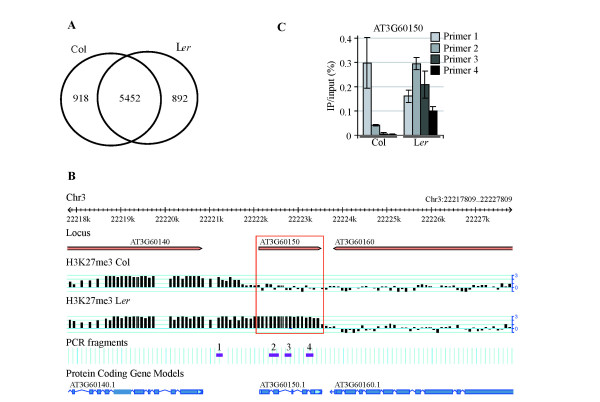
**Identification and validation of L*er*-specific H3K27me3 targets**. **(a) **Venn diagram of H3K27me3 targets identified in Col and L*er*. **(b) **GBrowse view of AT3G60150 as an example for a L*er*-specific H3K27me3 target. The red frame indicates the location of AT3G60150 and the differential H3K27me3 signal in Col and L*er*. **(c) **Validation of AT3G60150 by independent ChIP-PCR. ChIP-PCR fragment location is indicated in track 'PCR fragments' in (b). IP, immunoprecipitation.

To uncover the natural variation of H3K27me3 distribution within the species *A. thaliana*, the genome-wide distribution of H3K27me3 in two accessions, Col and L*er*, was profiled using chromatin immunoprecipitation (ChIP) followed by hybridization to whole-genome tiling microarrays (ChIP-chip). The original probes based on the TAIR6 *Arabidopsis *genome release were remapped to TAIR9 to ensure that unique, high quality probes were used to predict H3K27me3 targets in Col and L*er*. A visual analysis of the Col and L*er *H3K27me3 levels with GBrowse revealed that the majority of loci exhibited very similar H3K27me3 enrichment along chromosomes (Figure S1 in Additional file 1). Based on unique probes in Col after remapping, 6,370 and 6,344 H3K27me3 targets were identified in Col and L*er*, respectively (Figure 1a; Additional file 2). This number of H3K27me3 targets in Col is consistent with previous studies carried out on Col seedlings by different laboratories using various experimental platforms [26-29]. There are 5,452 H3K27me3 targets shared between Col and L*er*, indicating highly similar H3K27me3 profiles between the two accessions (Figure 1a).

### Differential H3K27me3-enriched genes are rare between Col and L*er*

The global comparison of targets between two accessions did not permit an evaluation of statistical significance and could indeed be fully explained by a false discovery rate inherent to genome-wide approaches. In addition, when using the same microarrays for two different *Arabidopsis *accessions, some amount of differential signals are to be expected due to sequence polymorphisms. The major sequence polymorphisms between Col and L*er *include presence/absence polymorphisms, copy number differences and single nucleotide changes (SNPs). Presence/absence polymorphisms lead to a signal being detectable in only one genome, which could be wrongly identified as a differentially methylated target. Copy number differences result in different cross-hybridization signals. SNPs lower the efficiency in hybridization and thus may result in a lower signal and false identification of differential targets.

To overcome these limitations, we tested each gene individually for differential signal. We used rank product statistics after calculating a median enrichment value over each gene body [30,31]. The analysis only included probes that mapped uniquely to both the Col genome and the published L*er *assembly (see Materials and methods) [32]. To identify genes enriched for H3K27me3 only in L*er*, a maximal number of two mismatches were permitted so that homologous genes with moderate sequence diversity in L*er *were retained. In contrast, for the identification of Col-specific H3K27me3 targets, no mismatch (*n *= 0) was allowed during remapping, which means only probes with perfect, unique matches in both accessions were included. This excludes false positives due to SNPs in Ler that lead to a lower signal of non-perfectly matching probes. As a consequence of the stringent remapping, approximately 25% of the genes annotated in TAIR9 and 10% of the H3K27me3 target genes detected by genome-wide target prediction in both Col and L*er *were removed from the potential target set due to a probe density that was too low to judge their methylation state in both accessions (Additional file 3). Setting a significance threshold for a proportion of false positives of 15% (pfp = 0.15), we identified 32 L*er*-specific and 11 Col-specific H3K27me3 targets (Table S3 in Additional file 1). Differential H3K27me3 occupancy in Col and L*er *was confirmed by quantitative PCR (qPCR) for 14 of 15 randomly chosen L*er*-specific H3K27me3 targets using independently prepared ChIP samples, confirming that the prediction is as reliable as expected at an observed false positive rate of 0.07 (Figure 1b,c; Figure S2A in Additional file 1). However, the Col-specific H3K27me3 targets showed inconsistent results between ChIP-chip and ChIP-PCR. This is most likely due to the difficulties associated with using a microarray designed for only one accession, Col. We therefore excluded Col-specific H3K27me3 targets from our further analysis (Figure S2B in Additional file 1).

### L*er*-specific H3K27me3 targets can be clustered into two groups based on their expression patterns

Previous studies showed that H3K27me3 target genes are lowly expressed in *Arabidopsis*, although a considerable number can reach high expression levels in specific tissues or conditions [25,26]. Two recent genome-wide comparisons of H3K27me3 distribution in specific tissues revealed a tendency for locally reduced H3K27me3 levels in tissues where the corresponding target genes are expressed [29,33]. In particular, H3K27me3 target genes expressed exclusively in either floral organs, the shoot apex or seeds often play a regulatory role in development and show an overrepresentation of transcriptional regulators [26,34]. In contrast, the Gene Ontology annotation for L*er*-specific H3K27me3 targets included no functions in development or transcriptional regulation (data not shown).

**Figure 2:**
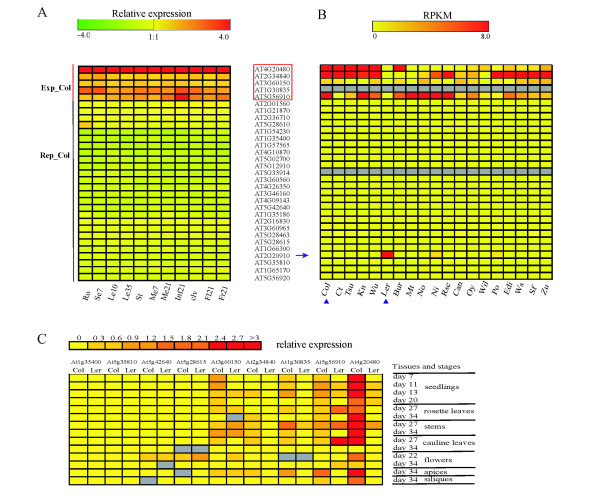
**Classification of L*er*-specific H3K27me3 targets based on their expression**. **(a) **Expression pattern of L*er*-specific H3K27me3 targets in Col in different tissues. The expression value was normalized to the mean of all genes within each sample. Five expressed L*er*-specific targets (Exp_Col) are framed in red. The developmental stages and tissues used for experiments are: roots at 7 days (Ro); seedlings, aerial parts, 7 days (Se7); expanding leaves, 10 days (Le10); senescing leaves, 35 days (Le35); stem, 2nd internode (St); vegetative shoot meristem, 7 days (Me7); inflorescence shoot meristem, 21 days (Inf21); whole inflorescences to floral stage 9, 21 days (Me21); whole inflorescences of *clavata3-7 *mutants, 21 days (clv); flowers, stage 15, 21+ days (Fl21); fruits, carpels stage 15, 21+ days (Fr21). The data were obtained from the *Arabidopsis thaliana *Tiling Array Express (At-TAX) resource [35]. **(b) **Expression pattern of L*er*-specific targets in 19 *Arabidopsis *accessions. Accessions indicated below the heatmap are Columbia-0 (Col), Catania-1 (Ct), Tsushima-0 (Tsu), Kaunas-0 (Kn), Würzburg-0 (Wu), Landsberg *erecta *(L*er*), Burren-0 (Bur), Martuba-0 (Mt), Nossen-0 (No), Hilversum-0 (Hi), Rschew-4 (Rsc), Canary Islands-0 (Can), Oystese-0 (Oy), Wilna-2 (Wil), Poppelsdorf-0 (Po), Edinburgh-0 (Edi), Wassilewskija-0 (Ws), San Feli-2 (Sf), Zurich-0 (Zu). AT2G20910 (arrow) did not express in Col but in L*er *and another three accessions. Gray indicates that the genes were not analyzable in the data-set, which was generated by the 19 Genomes Project [36]. RPKM, reads per kilobase per million mapped reads. **(c) **Expression of nine L*er*-specific H3K27me3 targets in Col and L*er *at different developmental stages or tissues. Expression was measured by qRT-PCR, and is plotted relative to the reference gene *AT1G13320*. Analyzed time points and tissues are as indicated. Expression was measured in two independent experiments with similar results.

To further characterize the L*er*-specific H3K27me3 targets, we analyzed their expression in Col, L*er *and other accessions. Expression patterns in different developmental stages and tissues in Col were explored using *Arabidopsis thaliana *Tiling Array Express (At-TAX) expression data, which have been generated by whole genome tiling microarrays [35]. Based on this analysis, L*er*-specific H3K27me3 targets clustered in two very distinct groups (Figure 2a). One group (termed Rep_Col) contained 26 genes and showed no expression in almost all included samples, despite the absence of the repressive mark of H3K27me3 in Col. A smaller group of five genes showed relatively high expression in almost all studied samples (termed Exp_Col).

We then further investigated the expression pattern of L*er*-specific H3K27me3 targets in seedlings of 19 *Arabidopsis *accessions as determined by RNA-seq [36]. The 19 accessions had been selected to represent the diversity within the species; Col and L*er *are relatively closely related within the group. Exp_Col and Rep_Col genes showed expression characteristics consistent with their class in seedlings of 19 *Arabidopsis *accessions (Figure 2b): four of five Exp_Col genes showed variable expression among accessions. The fifth gene, *AT1G30835*, a SADHU retroelement, was not analyzable in this dataset. However, natural variation in expression of *SADHU *TEs has been described earlier. *AT1G30835 *is highly expressed in Col and was considered to be silent in L*er *[37]. Notably, the remaining four genes were at their lowest expression in L*er *but only occasionally repressed among the other 18 accessions. All Rep_Col genes, except *AT2G20910*, showed constant repression in seedlings in all 19 accessions. *AT2G20910*, annotated as a pseudogene in Col, showed expression in seedlings of only two accessions in addition to L*er*. Expression data from seedlings cannot capture a tissue- or stage-specific expression pattern that is often shown by H3K27me3 target genes [26]. To better explore the expression pattern of L*er*-specific H3K27me3 targets at different stages and in different tissues, we directly measured the transcription level of nine L*er*-specific H3K27me3 targets in Col and L*er*. The nine targets included all five Exp_Col and four Rep_Col genes. Exp_Col genes showed a typical tissue or stage-restricted expression pattern commonly observed for H3K27me3 targets (Figure 2c). The four Rep_Col genes, on the other hand, were constantly repressed in both accessions at all time points analyzed irrespective of the absence or presence of H3K27me3 (Figure 2c). In conclusion, the expression pattern of L*er*-specific H3K27me3 targets in L*er *and Col confirmed that these can be categorized into two groups, one generally more active in the absence of the repressive H3K27me3 modification and the other transcriptionally repressed despite the absence of H3K27me3.

### L*er*-specific H3K27me3 targets are associated with distinct histone modifications in Col

**Figure 3:**
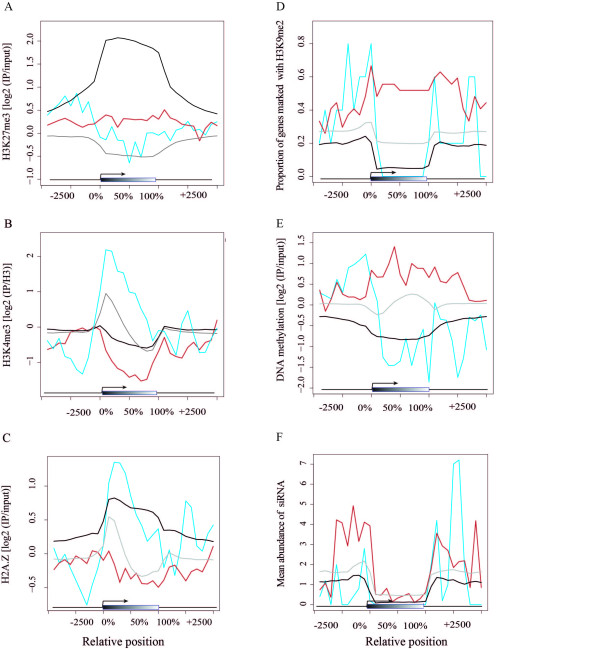
**Association of L*er*-specific H3K27me3 targets with chromatin modifications**. The degree of overlap with histone-modification positive regions was plotted for 10% length intervals along the gene body and for 500 bp sequence intervals for the 5-kb regions up- and downstream for Rep_Col (red), Exp_Col (blue), common H3K27me3 target (black) and non-H3K27me3 target genes (gray). The x-axis shows the relative position (gene body indicated by gray box), the y-axis represents mean signal of the respective mark (for (a,b,c,e)), proportion of genes overlapping with the mark (d) or normalized mean feature counts (f). **(a) **Enrichment of H3K27me3, **(b) **H3K4me3 [27], **(c) **H2A.Z [41], **(d) **H3K9me2 [42], **(e) **DNA methylation [42] and **(f) **association with 24-nucleotide siRNAs [62] of Rep_Col, Exp_Col, H3K27me3 targets and non-targets.

To evaluate how the expression pattern of L*er*-specific H3K27me3 targets accords with other histone modifications, we investigated the signal profiles of several active and repressive histone marks that have been mapped in Col seedlings. As expected, both Rep_Col and Exp_Col genes were depleted of H3K27me3 in Col whereas targets in general are highly H3K27me3-enriched, especially over the gene body (Figure 3a). Being consistent with their active expression in Col, Exp_Col genes were enriched for the active mark H3K4me3 (Figure 3b) [27]. In contrast, Rep_Col genes were depleted in this mark (Figure 3b). The H3K4me3 pattern of Rep_Col in Col was similar to that of H3K27me3 targets (Figure 3b). In contrast, in non-targets, the H3K4me3 enrichment peaked at the 5' end of the transcribed region as it did in the Exp_Col set.

H2A.Z has been implicated in multiple roles in divergent organisms [38,39]. It was proposed that the incorporation of H2A.Z could promote histone turnover and chromatin accessibility. In embryonic stem cells, H2A.Z is enriched at PcG target genes and necessary for lineage commitment [40]. In *Arabidopsis*, H2A.Z is preferentially enriched around the transcriptional start site and the 3' end of genes (Figure 3c) [41]. Unexpectedly, in *Arabidopsis *the mark also extended across larger regions into the gene bodies over H3K27me3 target genes. Exp_Col genes showed high H2A.Z signal at the transcriptional start site, thus behaving as most non-H3K27me3 genes. In contrast, Rep_Col were not labeled by H2A.Z in Col, thus representing a set distinct from both H3K27me3 target and non-target genes.

Besides H3K27me3, H3K9me2 and DNA methylation are associated with gene repression. In contrast to the euchromatic mark H3K27me3, these two marks are mainly located in constitutive heterochromatin [42,43]. To evaluate if these repressive histone modifications are associated with the Rep_Col set, the percentage of genes being marked by these repressive modifications was calculated from available data sets [42,43]. H3K9me2 frequently marked Rep_Col genes, both in gene bodies and their surrounding regions, while H3K27me3 targets, non-targets and Exp_Col showed much lower levels, especially within gene bodies (Figure 3d). We confirmed the change from H3K27me3 to H3K9me2 enrichment for a subset of L*er*-specific H3K27me3 targets by ChIP-PCR (Figure S3 in Additional file 1). Consistent with the enrichment of H3K9me2, DNA methylation was also found highly enriched at Rep_Col but depleted at Exp_Col and H3K27me3 target genes (Figure 3e). H3K9me2 occurrence and in particular non-CG DNA methylation are highly correlated with siRNAs of 23 to 24 bp length [13]. Interestingly, the gene bodies of the Rep_Col set were devoid of these siRNAs as were the gene bodies of all other compared categories. However, Rep_Col genes were frequently flanked by siRNAs at their 5' and 3' ends, indicating that the heterochromatic status of Rep_Col genes may be explained by flanking genomic features (Figure 3f).

### L*er*-specific H3K27me3 targets are often flanked by transposable elements in the Col genome

**Figure 4:**
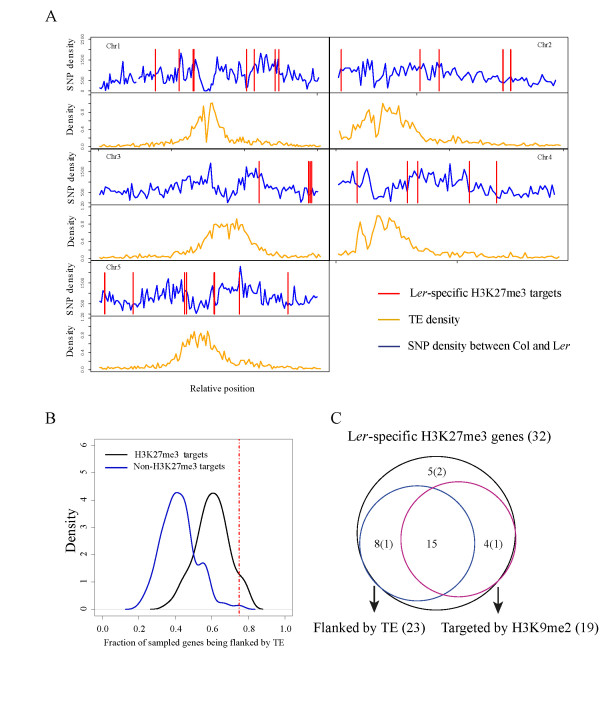
**Association of L*er*-specific H3K27me3 targets with TEs and H3K9me2**. **(a) **The distributions of L*er*-specific H3K27me3 targets relative to heterochromatic regions in chromosomes of Col. The red lines indicate the locations of L*er*-specific H3K27me3 targets; the blue lines indicate the SNP density between Col and L*er*; the brown lines indicate the density of TEs. **(b) **Density plot of fraction of genes flanked by a TE in a permutation test (sample size = 28, 200 drawings). The density is shown on the y-axis; the fraction of genes flanked by a TE for each sampling is shown on the x-axis. Non-H3K27me3 targets are indicated in blue, H3K27me3 targets in black. The dashed red line shows the fraction of 28 non-TE L*er*-specific H3K27me3 targets flanked by TEs in Col. **(c) **Association of L*er*-specific H3K27me3 targets with flanking TEs and the H3K9me2 mark. 19 L*er*-specific targets are H3K9me2 in Col, of which 15 are flanked by a TE. The number in brackets shows the number of annotated TE genes within the respective category.

TE-related sequences and their associated heterochromatic features are greatly enriched in pericentromeric regions where few actively transcribed genes can be found. To exclude the possibility that these genes were clustered within the pericentromeric regions, we mapped the chromosomal distribution of L*er*-specific H3K27me3 targets and found them mostly distributed around the euchromatic chromosome arms. The location of L*er*-specific H3K27me3 targets did not correlate with a general increase in TE or SNP density (Figure 4a). We therefore hypothesized that H3K9me2 at L*er*-specific H3K27me3 targets in Col might be recruited by TEs present in dispersed heterochromatin found in the chromosome arms. Indeed, the percentage of L*er*-specific H3K27me3 targets flanked by TEs in Col was significantly higher than that of non-H3K27me3 target controls (permutation test, *P *< 0.01; Figure 4b). However, H3K27me3 targets in general were also more likely to be flanked by TEs than non-target genes (Figure 4b). Notably, the absence or presence of TEs did not distinguish the Rep_Col from the Exp_Col set as three genes of the Exp_Col set were neighbors of a heterochromatin-associated TE (Table 1). In total, 23 L*er*-specific H3K27me3 targets were marked by H3K9me2 and 19 flanked by at least one annotated TE. Four genes are themselves annotated as TE genes (Figure 4c; Table S4 in Additional file 1). H3K9me2 targets are found significantly more frequently in the set of L*er*-specific H3K27me3 targets than in the set of all non-H3K27me3 targets (hypergeometric test, *P *= 1e-07).

**Table 1:** L*er*-specific H3K27me3 targets adjacent to TEs polymorphic between Col and L*er*

Left_TE	Ler-specifc H3K27me3	Group	Right_TE	TE family	PCR size in Col	PCR size in Ler	Confirmed target site duplication
AT2G16820	AT2G16830	Rep_Col	AT2G16832	Mutator-like	NA	NA	NA
AT2TE65230	AT2G34840	Exp_Col	NO	DNA/MuDr	2.8 kb	1.8 kb	ATTTG
NO	AT4G09143	Rep_Col	AT4G09146	LTR/Copia	NA	NA	NA
Node	AT5G35810	Rep_Col	AT5G35820	LTR/Copia	6.6 kb	1.0 kb	ATACCT
NO	AT5G42640	Rep_Col	AT5G42645	LTR/Copia	7.9 kb	3.0 kb	CCGCA
NO	AT4G20480	Exp_Col	AT4G20490	LTR/GYPSY	NA	NA	NA
AT4G10865	AT4G10870	Rep_Col	NO	AT4G10880 is not a TE	NA	NA	NA
AT2G01550	AT2G01560	Rep_Col	NO	Non-LTR retrotransposon (LINE)	NA	NA	NA
NO	AT3G60560	Rep_Col	AT3G60565	LTR/Copia	NA	NA	NA
AT5TE82820	AT5G56920	Rep_Col	AT5TE82825	left:DNA/AtREP10D; right:RC/Helitron	3.3 kb	2.0 kb	ATTAAGTAA
NO	AT5G56910	Exp_Col	AT5TE82820	DNA/RP1-AT	2.1 kb	1.8 kb	no
AT2TE37940	AT2G20910	Rep_Col	NO	DNA/MuDR	NA	NA	NA
NO	AT1G66300	Rep_Col	AT1TE81190	RC/Helitron	NA	NA	NA

NA, not available.

### TE insertions in Col and local genome rearrangements were found at a majority of H3K27me3 variant loci

**Figure 5:**
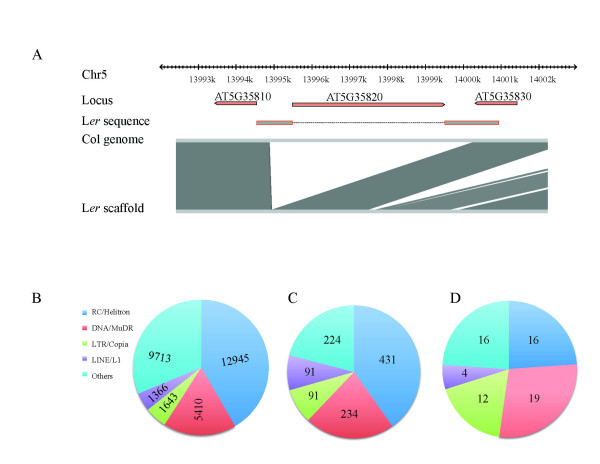
**Characteristics of TEs present in Col but absent in L*er***. **(a) **TE flanking a L*er*-specific H3K27me3 target (AT5G35810) is missing in L*er *assembly. The scale shows the sequence coordinates in the Col genome. The red bars show the gene models of *AT5G38510*, the annotated TE gene *AT5G38520 *flanking it and the following protein-coding gene *AT5G35830*. The short gray bars with red frame show sequences present in both genomes according to the sequencing of PCR products; the dashed gray line between short gray bars shows the sequence that is present in Col but missing in L*er*. Pictogram below shows sequences that were aligned between the Col genome and L*er *scaffold (in gray); white regions could not be aligned. **(b) **The distribution of different types of TEs in the Col genome. **(c) **Distribution of different types of TEs that are present in Col but absent in L*er*. **(d) **Distribution of TEs present in Col but absent in L*er *and that neighbor H3K9me2 targets.

The overrepresentation of TEs as neighbors of H3K27me3 target genes could create a higher imminent risk of H3K27me3 genes to be taken over by heterochromatin spreading. Such heterochromatic invasion could be stochastic and lead to accession-specific variation or could be explained by a variant propensity of specific TE families to spread in one accession but not the other. Alternatively, absence/presence polymorphisms of TEs could explain the epigenetic variation. To distinguish between these possibilities, we evaluated whether TEs that neighbor L*er*-specific H3K27me3 targets in Col are also present in the L*er *genome. A whole genome alignment between the Col reference genome and the L*er *draft genome [32] was carried out to extract sequences of L*er*-specific H3K27me3 targets and their surrounding regions (5,000 bp up-/downstream of each gene body) in the two genomes. In 11 out of 23 cases, TEs flanking L*er*-specific H3K27me3 targets were missing in the L*er *draft genome (Table 1). An example of a clean absence/presence polymorphism is AT5G35820, which is missing in L*er *while the flanking neighbors AT5G35810 (L*er*-specific H3K27me3 target) and AT5G35830 (common H3K27me3 target) are highly similar between the two accessions (Figure 5a; Figure S4 in Additional file 1). For intact polymorphic TEs, it is possible to distinguish between insertion and excision events by analyzing the target site signature. We selected five polymorphic, apparently intact TEs for verification by PCR amplification and Sanger sequencing (Table 1). The data indicate that the L*er *draft genome correctly identified the polymorphisms, which were in four cases more likely explained by a TE insertion in Col as opposed to an excision in L*er *based on the detection of target site duplications in Col but not L*er*. Interestingly, in two cases polymorphic TEs were linked to Exp_Col genes, indicating that the insertion of a TE was not sufficient to predict heterochromatic invasion.

The low number of L*er*-specifc H3K27me3 genes precludes a significant statement about whether certain transposons are more likely to cause heterochromatic invasion. We therefore investigated genome-wide if certain polymorphic TEs were more likely to spread heterochromatin into neighboring protein coding genes. To do this, the polymorphic regions identified by Schneeberger *et al.*[32] were annotated for the presence of TEs in Col. Compared to all TE classes, LTR/Copia and LINE elements were most polymorphic between Col and L*er*, considering presence in Col and absence in L*er *(Figure 5b,c). For 968 inserted TEs, 965 flanking protein-coding genes without TE insertion directly within the gene were identified in Col. Among these 965 protein-coding genes, 89 were found to be labeled with H3K9me2 [42]. Thus, protein-coding genes flanking Col-specific TEs are more frequently marked by H3K9me2 than expected on the basis of targets in the whole genome (hypergeometric test, *P *= 2 e-11). Compared with the distribution of all inserted TEs, the LTR/Copia family of retrotransposons was overrepresented among all TEs flanking H3K9me2-targeted protein coding genes (hypergeometric test, *P *< 0.005). Indeed, all L*er*-specific H3K27me3 genes that had acquired an LTR/Copia neighbor belonged to the heterochromatic Rep_Col set (Table 1). We conclude that in the *Arabidopsis *accession Col, TEs of the LTR/Copia family have a strong ability to spread their H3K9me2. Interestingly, the family also showed most differential insertions in Col as compared to L*er*, indicating that transposition history and heterochromatic spreading could be correlated (Figure 5a,b).

### H3K27me3 is stably inherited in reciprocal F1 hybrids in an allele-specific manner

**Figure 6:**
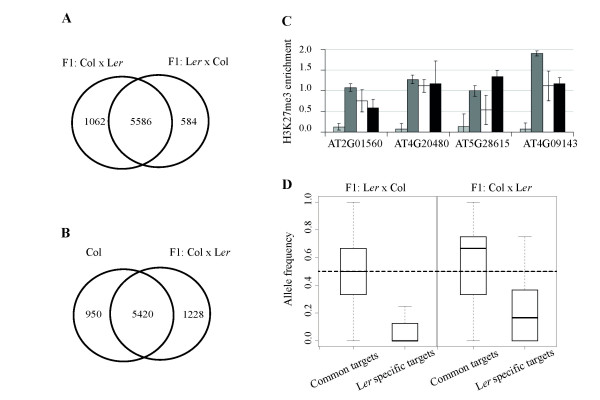
**Parental inheritance of H3K27me3 in reciprocal F1 hybrids**. **(a) **Venn diagram comparing H3K27me3 targets identified in Col × L*er *and L*er *× Col F1 hybrids by ChIP-Seq. **(b) **Venn diagram comparing H3K27me3 targets identified in Col by ChIP-chip and in Col × L*er *F1 hybrids by ChIP-Seq. **(c) **The H3K27me3 enrichment of four L*er*-specific H3K27me3 targets in Col (light gray), L*er *(dark gray), Col × L*er *(white) and L*er *× Col (black) F1 hybrids measured by ChIP qPCR. The enrichment was calculated relative to the enrichment at *FLOWERING LOCUS T*. Error bars represent standard error of one representative experiment. **(d) **Allele-specific H3K27me3 in reciprocal hybrids of Col and L*er*. Twenty-two SNPs for 12 L*er*-specific H3K27me3 targets in the L*er *× Col F1 hybrids and 35 SNPs for 15 L*er*-specific Col × L*er *F1 hybrids were used for allele frequency calculation. The dashed lines show the value of 0.5, which is the expected allele frequency value for heterozygous genes if both alleles are equally H3K27 tri-methylated.

Heterochromatin spreading is thought to depend, at least partially, on the recruitment of chromatin modifying complexes by siRNAs and the count of siRNAs was increased in the flanking regions of Rep_Col genes (Figure 3f). In the current model, the action of siRNAs is thought to occur in *cis *and *trans*, which could lead to a loss of the H3K27me3 mark from the L*er *allele in F1 hybrids, if these siRNAs were provided by the Col allele. We performed H3K27me3 profiling using the ChIP-Seq technique in the F1 generation of reciprocal hybrids between Col and L*er *(Col × L*er *and L*er *× Col, ♀ × ♂, respectively). The sequencing reads were mapped to the TAIR9 reference genome, permitting a mismatch of 3 to allow for small nucleotide polymorphisms in L*er*. We used reads from sonicated, non-precipitated chromatin as background to identify 6,648 and 6,170 H3K27me3 targets in the F1 of Col × L*er *and L*er *× Col, respectively (Additional file 2). These showed an overlap of 5,586 targets (Figure 6a). Of 6,370 H3K27me3 targets in Col, 5,420 were also detected in the F1 of Col × L*er *(Figure 6b). The overlap is similar to that observed between different ChIP-chip data sets and in the range of the expected false discovery rate.

Of the L*er*-specific H3K27me3 target genes, approximately half were still positive in both F1. To test whether the loss of H3K27me3 in the hybrids was due to *trans *regulation or an artifact caused by the expected reduced number of reads if those were generated by a single H3K27me3 positive allele, we chose four genes to verify their H3K27me3 state in F1 by ChIP-PCR. All tested loci were still H3K27me3 positive in both F1 groups (Figure 6c).

Targets that were detected as positive in the F1 ChIP-seq dataset allowed us to calculate the allele frequency using known SNPs between Col and L*er *[32]. In both F1 hybrids, the median allele frequency for L*er*-specific H3K27me3 targets is 0 for the Col allele, but 0.5 for common targets (Figure 6c). The allele frequencies of random samples from common targets were significantly different from those observed for L*er*-specific H3K27me3 targets (Permutation test, *P *< 0.001). In conclusion, the H3K27me3 mark showed clear allele-specificity in reciprocal hybrids and heterochromatic invasion in *trans *was not observed.

## Discussion

### The distribution of H3K27me3 target regions is almost invariant between Col and L*er*

Previous studies established that throughout development, the presence of H3K27me3 represents the default state for the majority of targets since only a small number of genes are differentially modified in different tissues and during the embryo-to-seedling transition [29,44]. In the developmental context, the loss of H3K27me3 in particular developmental stages or tissues is usually correlated with an increased expression of targets. Several thousands of genes show variable gene expression between different accessions of *A. thaliana *[45,46]. It was previously speculated that H3K27me3 played a role in allele-specific expression since, in F1 hybrids, differentially *trans*-regulated genes were depleted in H3K27me3 targets, whereas *cis*-regulated genes were slightly enriched [46]. The last trend was even more strongly observed in intraspecifc F1 hybrids between *A. thaliana *and *Arabidopsis lyrata*, but only for genes that were more highly expressed from the *A. lyrata *allele [47]. Our study established that most H3K27me3 targets are shared between seedlings of Col and L*er *and that the small number of differential H3K27me3 targets cannot mechanistically explain global expression differences between accessions. A possible caveat of this conclusion is that subtle differences in H3K27me3 levels at shared targets may not be resolved by the ChIP-chip method employed and have therefore escaped our attention.

Our data are consistent with a previous study in which H3K27me3 targets were compared between the accessions CVI, C24 and Col [28]. However, the methods to identify differential targets differed between the studies, leading to a considerably higher number of differential H3K27me3 targets identified in the CVI, C24 and Col comparison. As the study by Moghaddam *et al.*[28] did not include a statistical test to evaluate differential targets, it is likely to have generated a considerable number of false positives. In contrast, our stringent remapping of microarray probes to both the Col genome and the L*er *assembly could result in an underestimation of differential targets as highly polymorphic and very similar duplicated regions were excluded from the potential target list (Additional file 3). In particular, this exclusion could be the reason of our failure to detect genuine Col-specific H3K27me3 target regions. The 32 L*er*-specific H3K27me3 targets therefore represent a high confidence gene set that allowed us to pinpoint structural features that could explain the observed epigenetic differences.

### *Cis*-effect of H3K27me3 inheritance in reciprocal hybrids of Col and L*er*

It is not entirely clear by which mechanisms PcG-complexes are recruited to their target sites, although there is mounting evidence that longer non-coding RNA transcripts are involved [24,48]. Such RNAs could act in *trans *to recruit H3K27me3. L*er*-specific H3K27me3 targets contained no clear candidates of *de novo *recruitment of H3K27me3, which made it unlikely that H3K27me3 would be recruited to the Col allele in F1 hybrids. siRNAs generated by TEs flanking L*er*-specific H3K27me3 targets could also act in *trans *to initiate heterochromatin formation at the L*er *allele in F1 hybrids. Such a process could lead to the generation of stable epi-alleles. Our ChIP-seq data excluded any possibility of *trans*-acting effectors, since the inheritance of H3K27me3 was strictly allelic. This is consistent with a previous study that compared histone methylation patterns in hybrids between rice cultivars [49].

### Expression of L*er*-specific H3K27me3 targets is consistent with their chromatin states

Although the expectation was that the loss of the repressive H3K27me3 mark correlated with a gain in expression, only the small set of Exp_Col genes substantiated this expectation. The larger Rep_Col gene set was neither expressed in a variety of tissues in Col nor in the seedling stage of 18 additional *Arabidopsis *accessions (Figure 2).

The expression pattern of L*er*-specific H3K27me3 targets can be explained through an analysis of available chromatin modification data in Col. Based on a global analysis of 12 chromatin modifications, Roudier *et al.*[18] defined four chromatin states, CS1 to CS4, of which CS1 is associated with active expression of genes, whereas CS2 to CS4 are condensed chromatin states with little or no gene expression [18]. CS1 is characterized by the active histone modifications H3K4me3, H3K36me3 and H3K9me3, the latter a euchromatic mark in *Arabidopsis *but heterochromatic in animals. CS2 is defined by the repressive mark H3K27me3 and CS3 by the silencing mark H3K9me2 as well as increased DNA methylation. CS4 is ill defined by chromatin modifications as none of the 12 marks seems enriched. While common H3K27me3 targets were clearly CS2, we found the CS1 attribute H3K4me3 enriched at Exp_Col genes, whereas CS3 marks were enriched at Rep_Col genes (Figure 3).

### Chromatin changes leading to differential H3K27me3 targeting

In the expression comparison between seedlings of different accessions, all Exp_Col genes were at their lowest expression state in L*er*, but showed only occasional absence of expression among the remaining 17 accessions. For two Exp_Col genes, AT3G60150 and AT2G34840, H3K27me3 invasion from flanking regions could explain the gene-specific H3K27me3 enrichment in L*er*.

For most Rep_Col genes, H3K9me2 seems to be the derived state based on the following observations. The presence of siRNA signatures at the flanks rather than bodies of Rep_Col genes indicated that heterochromatic regions in the vicinity of Rep_Col genes play a functional role in the change from CS2 to CS3 chromatin. In Col, Rep_Col genes were frequently flanked by TEs, which could generate these siRNAs, but this feature did not distinguish Rep_Col genes from H3K27me3 targets in general (Figure 4). However, in several cases, flanking TEs were missing in L*er*, suggesting that their insertion in Col changed the chromatin from CS2 to CS3 (Figure 5; Figure S3 in Additional file 1). In several cases, analysis of target site duplications supports the insertion of the TE in Col as opposed to an excision in L*er*. It seems likely that siRNAs produced by, or targeted to, the inserted TEs recruit heterochromatic modifications such as DNA methylation and subsequently H3K9me2, which then spread into the adjoining H3K27me3 target gene. Since the CS2 and CS3 chromatin is mutually exclusive, such heterochromatin invasion would lead to a loss of H3K27me3 [18,25].

It is unclear why the Rep_Col genes were invaded by heterochromatin whereas, in general, H3K27me3 target genes appear to form a barrier to heterochromatin spreading given the enrichment of TEs in their neighborhood (Figure 4). Two scenarios may explain these observations. First, particular families/classes of flanking TEs could be more invasive than others and thus particularly enriched in the vicinity of Rep_Col genes. In mammals, a different tendency of TEs to spread H3K9me3 to nearby regions has been described [50]. Indeed, we found an over-representation of heterochromatic protein-coding genes in the vicinity of LTR/Copia TEs that are polymorphic between Col and L*er *(Figure 5). Absence of selection could be a second scenario to explain heterochromatic spreading only for a small subset of TE-flanked H3K27me3 targets. If expression of Rep_Col genes is not required under any circumstances neither in Col nor L*er*, complete silencing by the heterochromatin pathway instead of repression by the PcG pathway has no consequences for the plants. Interestingly, in two cases the Exp_Col genes flanked heterochromatic TEs that were absent in L*er *(Table 1). Possibly, in these situations high expression and the corresponding CS1 chromatin have evolved to form a strong barrier against invasive heterochromatin. Schmitges *et al.*[11] proposed that H3K4me3 acts as a barrier for the deposition of H3K27me3, and this could apply to repressive marks in general. CS1 chromatin could have been selected for because residual expression of these Exp_Col genes gave a fitness advantage, but their role could also be more passive to provide a functional chromatin barrier because adjacent genes were important for plant survival. Alternatively, it may have been the TE insertions *per se *that caused activation of a flanking target. It has been shown in maize and rice that transposon insertions can have positive effects on gene expression through disruption of native promoter regulation or introduction of new regulatory elements [51,52].

Although polymorphic TEs may explain the majority of L*er*-specific H3K27me3 targets, there are some limitations. L*er*-specific H3K27me3 targets are not always flanked by TEs and the corresponding TEs are not always missing in L*er*, indicating that polymorphic TEs are not the only cause of variation in H3K27me3. Possibly, in at least some cases serendipitous recruitment of H3K27me3 in one and H3K9me2 in the other accession to genes not expressed in either accession resulted in differential labeling.

## Conclusions

Intraspecific variation in H3K27me3 is rare and an unlikely cause for differential expression between accessions. A small, high-confidence set of L*er*-specific H3K27me3 targets allowed pinpointing structural features that explain intraspecific variation for this epigenetic mark. Insertion of TEs at the flanks of target genes seems the major cause of H3K27me3 loss through heterochromatic invasion. In particular, retrotransposons of the LTR/Copia class were linked to H3K9me2-marked protein coding gene loci, indicating that these TEs were particularly aggressive towards their neighbors.

## Materials and methods

### Plant material and growth conditions

*A. thaliana *accessions Col and L*er *and their hybrids were grown under long-day conditions (16 hours light, 8 hours dark) for 10 or 11 days at 20°C on Murashige and Skoog medium supplemented with 1% sucrose after stratification at 4°C for 2 to 4 days to synchronize germination. Light was provided by fluorescent tubes. For intraspecific crossing, Col and L*er *plants were grown on soil under long-day conditions. Five flower buds on the primary shoot and two side shoots were emasculated and manually cross-pollinated, while all other flower buds were removed. The seeds of each genotype were pooled and the success rate of the crosses was determined by PCR using primers that detected an insertion between L*er *and Col (forward, 5'-CTGGAGATCATCCAACAAAGG-3'; reverse, 5'-GGCAATGGAATGGGCTGGTC-3'). Seed pools with less than 10% maternal contamination were used in the F1 hybrid studies.

### ChIP experiments

ChIP experiments were performed as described [53] except that chromatin was sonicated with a BioRuptor from Diagenode (Liège, Belgium) for 10 times 30s at high setting with 60 s intermittent cooling in ice-water. A DNA fragment size of 300 to 1,000 bp was controlled by running an aliquot of de-crosslinked and purified DNA on 1.5% agarose gels. The following antibodies were used in immunoprecipitations: anti-rat IgG (R9255, Sigma; St. Louis, MO, USA), anti-H3K27me3 (07-449, lot number DAM 1662421, Millipore; Temecula, CA, USA), and anti-H3K9me2 (pAB-060-050, lot number 90-0041, Diagenode; Liège, Belgium). Specificity of antibodies was found to vary between lots and was therefore confirmed for each new lot by western blot against a dilution series of modified peptides (Figure S5A in Additional file 1). Furthermore, cross-hybridization of H3K27me3 antibodies with H3K27me1 signal was excluded by probing Ler-specific H3K27me3 targets with antibodies specific for H3K27me1 (pAB-045-050, lot number A116-00342, Diagenode; Liège, Belgium) (Figure S5B in Additional file 1). A very low signal was detected in anti-rat IgG-antibody precipitations and was subtracted as background. qPCR data are shown as fold enrichment of the input, or fold enrichment of input and compared to the fold enrichment of the input for the *FLOWERING LOCUS T *gene as indicated; the error bars represent standard error of three technical replicates. At least two independent biological replicates were performed for each experiment and a representative one is shown. Primers used for qPCR are described in Table S5 in Additional file 1.

### ChIP-chip and ChIP-Seq experiments

ChIP-chip experiments were carried out as described elsewhere using 10- or 11-day-old seedlings harvested at ZT16 [53]. DNA samples were amplified using a linker-mediated PCR and hybridized to two-color microarrays from Roche-NimbleGen (Madison, WI, USA); input samples were hybridized as reference. Two biological replicates were hybridized per accession. For ChIP-Seq, 80% of a ChIP experiment precipitated with H3K27me3 antibodies was used to prepare libraries using the ChIP-Seq library preparation kit from Illumina (number 11257047) according to the manufacturer's instructions. Each library was loaded on two lanes of the Illumina Genome Analyzers IIx to obtain single end 34-mer reads. The sonicated input of a chromatin sample from Col was used as background reference and loaded only on one lane for sequencing.

### ChIP-chip data analysis

ChIP-chip data were analyzed using the custom R package ChIPR [31] that integrates the RINGO package [54]. Probes were remapped to the Col and L*er *genomes using BWA 0.5.9 [55] allowing maximal two mismatches. To predict H3K27me3 targets in Col and L*er*, only probes uniquely mapped to the Col TAIR9 genome with an edit distance of two nucleotides to the second best matching site were used. This step removed probes that were likely to hybridize to more than one region. A descriptive ndf-file was created that contained only the reannotated probes. Following the ChIPR pipeline, loess normalization was applied within arrays then the normalization method 'scale' was used between arrays to remove inconsistencies. Positive probes were identified by the method implemented in RINGO and all positive probes separated by gaps smaller than 300 bp were combined into ChIP-enriched regions. Genes covered by these regions for at least 30% of their gene body and at least 300 bp or minimally 1,000 bp (for very long genes) were defined as H3K27me3 targets.

To predict differentially H3K27me3 enriched genes between the accessions, only probes uniquely mapped to the Col genome and the published L*er *scaffolds were used. For each annotated gene in TAIR9, a median value was determined per replicate and accession based on all probes that covered the gene body. Genes with less than three uniquely mapped probes or less than 40% coverage by probes were removed from the analysis. Genes that were not H3K27me3 targets in the global target analysis of either genome were also excluded to ensure that the Col- and L*er*-specific H3K27me3 targets have high H3K27me3 signal at least in one accession (Table S3 in Additional file 1). The R package RankProd [56] was used to define the most differentially labeled genes between Col and L*er*. Genes significant at a proportion of false predictions (pfp) smaller than 0.15 were regarded as differentially enriched in one accession.

### ChIP-Seq data analysis

Reads from two Illumina GAIIx lanes for each sample were merged and mapped to the Col reference genome (TAIR9) using BWA 0.5.9 [55]. The maximal edit distance was three including a maximal gap of one. Low quality sequences at read ends were trimmed with BWA while mapping. Reads mapping to the genome were sorted according to the Col TAIR9 genome coordinates using SAMtools. [55]. Reads mapped to identical positions in the genome were cleaned with Picard MarkDuplicates [57] by keeping one copy to avoid PCR artifacts. H3K27me3 enriched targets in the F1 generation of Col × L*er *and L*er *× Col were predicted with SICER V1.03 using the chromatin input from Col as background [58]. Only uniquely mapped reads were used for prediction of peaks. Peaks separated by gaps smaller than 200 bp were merged. In hybrids, genes with at least 20% and 200 bp, or 800 bp (for very long genes) covered by peaks were defined as H3K27me3 targets. A short reads pileup format file was generated by SAMtools based on the non-redundant reads. This file shows which and how many reads pile up at genomic coordinates. The pileup file was used for detection of allele-specific enrichment for H3K27me3. SNP data between Col and L*er *were downloaded from the 1001 Genome Project [39]. The allele frequencies of L*er*-specific and common H3K27me3 targets were calculated based on the SNP data and the short reads pileup format file. SNP positions with less than three uniquely mapped reads were excluded. Reads that were not the same as the reference Col nor the L*er *allele at SNP positions were also excluded. To test whether there is a high probability to observe the allele frequency of L*er*-specific targets also in the common H3K27me3 target list, the same number of SNPs as analyzed in the L*er*-specific targets were drawn from all SNPs in H3K27me3 targets for 100,000 times. The allele frequency was calculated for each randomization. This resulted in a *P*-value <0.00001 being calculated for both reciprocal hybrids.

### Sequence comparison between Col and L*er*

The *Arabidopsis *Col genome (TAIR9) was used as reference genome. L*er *genome data were obtained from the 1001 Genomes data center [59]. The whole genome alignment tool MUMmer was used to align all scaffolds of L*er *to the Col reference sequence. The alignment was performed following the instructions for 'Mapping a draft sequence to a finished sequence' [60]. The parameter setting used was 'nucmer --mum -b 1000 -l 35 -c 80 -f --prefix = outputFolder referenceSquence *LerassemblySequence*'. With such a setting, first only anchors that were unique in both reference and query were allowed for alignment. nucmer further extended alignments across high diversity regions until reaching maximally 1,000 edit distance. If the diverged regions or indels are larger than 1,000 bp, the alignment will break. Finally we restricted the alignment to match the forward strand of the query.

To check whether the transposable elements flanking L*er*-specific H3K27me3 target genes in the Col genome exist also in the L*er *genome, the respective sequences of gene bodies and their up/downstream regions were extracted from both genomes using a custom R script and aligned again with MUMmer. The sequences and the alignment result visualized with Artemis Comparison Tool [61] for one example gene are shown in Figure 5.

The detected deletion of transposable elements was further tested for five L*er*-specific H3K27me3 loci by sequencing PCR products spanning the respective region. Genomic DNA from Col and L*er *was used as template (PCR primer list in Table S5 in Additional file 1). PCR was performed using Phusion Taq (M0530 S; NewEngland BioLabs (Ipswich, MA, USA)) with the high fidelity buffer. PCR product size was determined on 1% agarose gels. PCR products were either purified by polyethylene glycol precipitation or directly from the gel using a GelExtraction Kit (Machery-Nagl Düren, Germany) before Sanger sequencing. Sequences obtained from L*er *were aligned to the Col genome (TAIR10) using BLAST.

### Expression analysis

Whole seedlings grown on plates were harvested for total RNA extraction at day 7, 10 and 12. The aerial part of soil-grown plants was collected on day 13 and 20 and tissue-specific samples (rosette and cauline leaves, stem, open flower, apex enriched tissue and silique) were obtained at days 27 and 34 to extract total RNA with the RNeasy Mini kit (Qiagen, Hilden, Germany). Five micrograms of RNA was DNaseI treated using the DNA-free kit (Ambion Austin, TX, USA) prior to cDNA synthesis with SuperScript II Reverse Transcriptase (18064-014, Invitrogen Carlsbad, CA, USA). Quantitative reverse-transcribed (qRT)-PCR was performed using a Roche LightCycler (Mannheim, Germany) and EVA Green dye detection (Biotium, Hayward, CA). PP2A (AT1G13320) was used as reference gene. Primers are listed in Table S5 in Additional file 1.

### Primary accession

ChIP-chip and ChIP-Seq data reported in the manuscript are available at ArrayExpress under series accession number E-MTAB-749 (in accordance with MIAME guidelines) and E-MTAB-1043, respectively.

## Abbreviations

Bp: base pair; ChIP: chromatin immunoprecipitation; ChIP-chip: chromatin immunoprecipitation followed by microarray hybridization; ChIP-PCR: chromatin immunoprecipitation followed by PCR; ChIP-seq: chromatin immunoprecipitation followed by high throughput sequencing; Col: Columbia; HMT: histone methyl-transferase; CS: chromatin state; H3K4me3: histone H3 tri-methylated at lysine 4; H3K9me2: histone H3 di-methylated at lysine 9; H3K27me3: histone H3 tri-methylated at lysine 27; L*er*: Landsberg *erecta*; PcG, Polycomb group; PCR: polymerase chain reaction; PRC: Polycomb repressive complex; qPCR: quantitative PCR; qRT-PCR: quantitative reverse-transcribed PCR; siRNA: small interfering RNA; SNP: single nucleotide polymorphism; TAIR: The *Arabidopsis *Information Resources; TE: transposable element.

## Authors' contributions

JR performed the experiments, XD analyzed the data, and both evaluated and interpreted the data. UG, JE and FH made important suggestions to improve the analysis and interpretation of data. FT conceived the study; HS and FT coordinated the research. XD, JR, HS and FT wrote the manuscript. All authors read and approved the final manuscript.

## Supplementary Material

Additional file 1 **Supplementary Figures S1 to S5, Tables S3 to S5 and Supplemental Methods**. Click here for file

Additional file 2: Table S1 Supplemental Table 1. H3K27me3 target gene list of Col, Ler, F1 hybrids Col x Ler and Ler x Col. Click here for file

Additional file 3: Table S2 Supplemental Table 2. Genes that were removed from the analysis after stringent remapping of probes to Col and Ler scaffolds. Click here for file
