# Supplementary material for: Natural variation of H3K27me3 distribution between two Arabidopsis accessions and its association with flanking transposable elements
Source: Genome Biol. 2012 Dec 19;13(12):R117. doi: 10.1186/gb-2012-13-12-r117 (PMC4056368; doi:10.1186/gb-2012-13-12-r117)
Supplement: Additional file 1 — Supplementary Figures S1 to S5, Tables S3 to S5 and Supplemental Methods. [file gb-2012-13-12-r117-S1.PDF]

Supplemental Figures, Tables and Methods

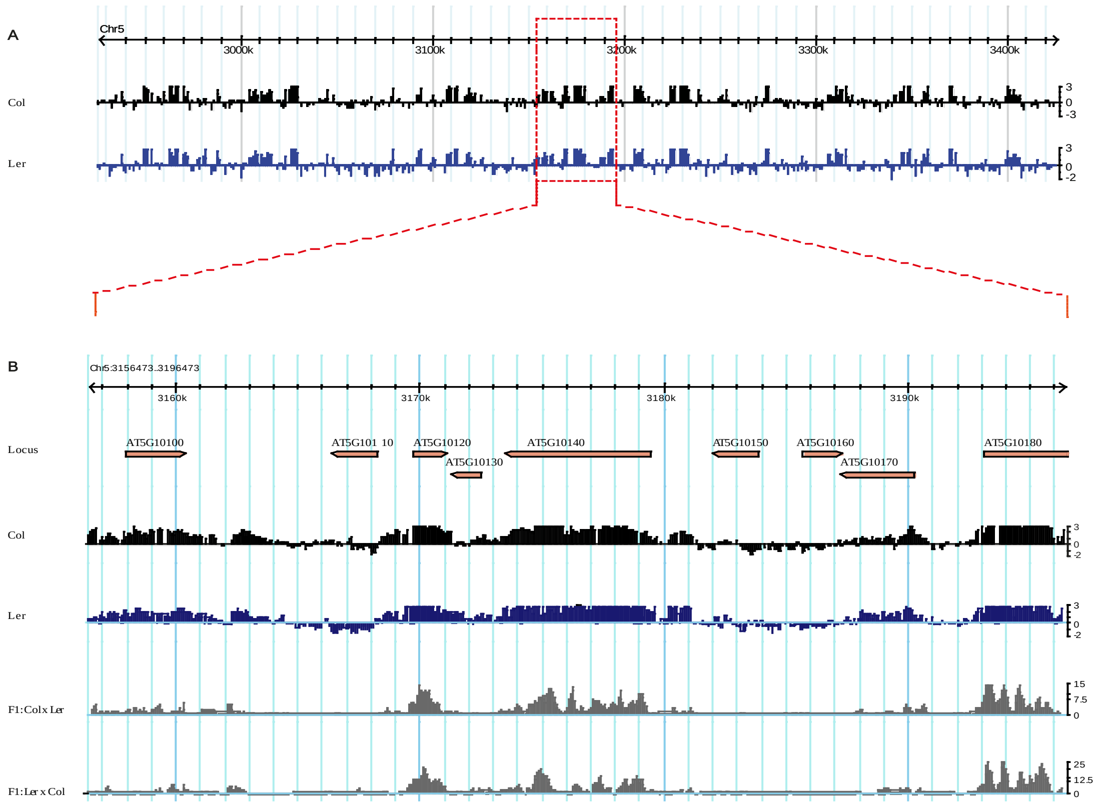

Supplemental Figure 1. H3K27me3 profiles in Col and L*er* are similar.

(**A**) GBrowse overview of a representative 0.5 Mb region on chromosome 5. Tracks show scaled mean log2 values (IP/INPUT) for each probe from two replicate ChIP-chip microarrays for each Col and L*er*.

(**B**) Zoom into a 30-kb region of chromosome 5 including representative gene AT5G10140. The track 'Locus' shows genes (red boxes) annotated in TAIR9. H3K27me3 profiles in Col (black) and L*er* (blue) are as in (A), reciprocal F1 hybrids (gray) are analyzed by ChIP-seq. Data show 300 bp extended unique reads.

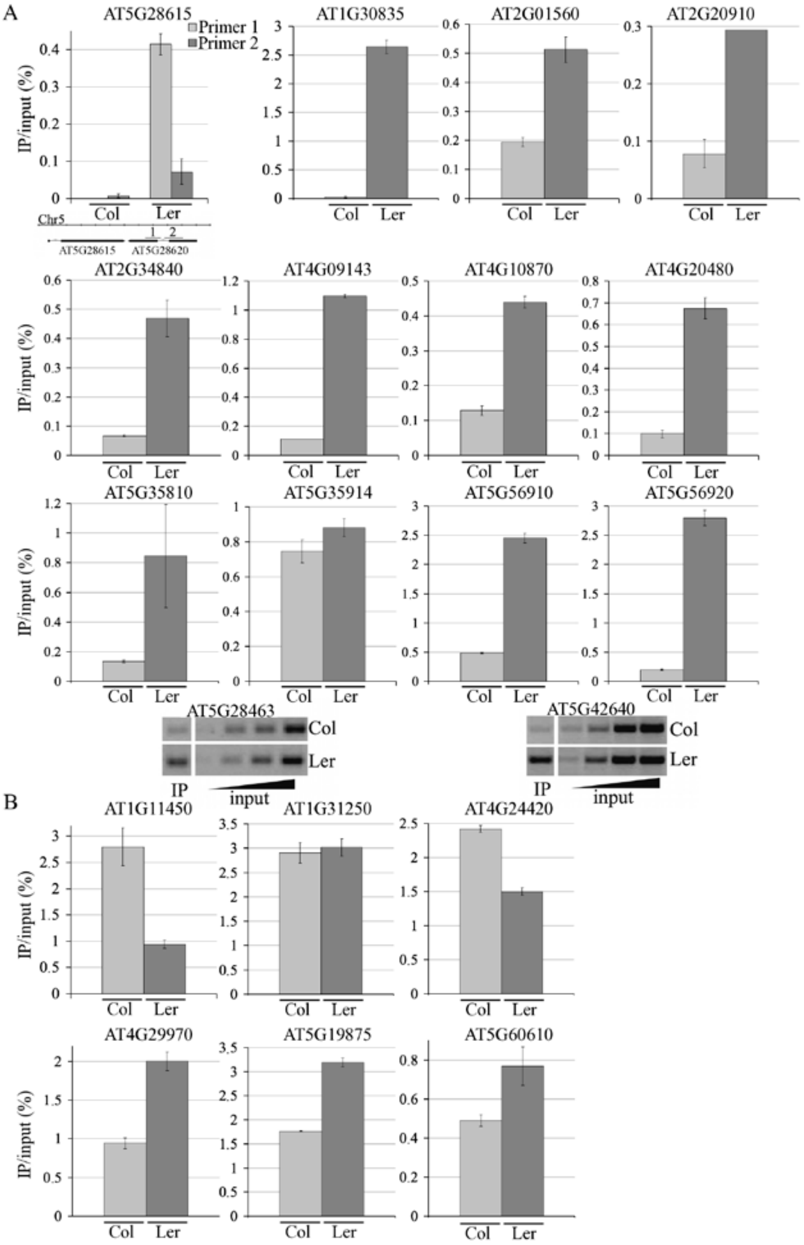

Supplemental Figure 2. **Confirmation of L*er* specific H3K27me3 targets and Col specific H3K27me3 targets via ChIP-PCR.** Tissue from ten day old whole seedlings was used to perform a ChIP for H3K27me3 (**A** and **B**). Random sets of L*er* specific H3K27me3 targets (**A**) and Col specific H3K27me3 target genes (**B**) were chosen to confirm the enrichment for H3K27me3 using qPCR or a semi-quantitive agarose gel approach. Data are shown as ChIP enrichment per input chromatin. For all loci except AT5G28615 light gray bars represent the result for Col and dark gray bars for L*er*. For AT5G28615 two PCR fragment positions are indicated in the gene model below the diagram. Data show the mean of one representative experiment with SE based on three technical replicates.

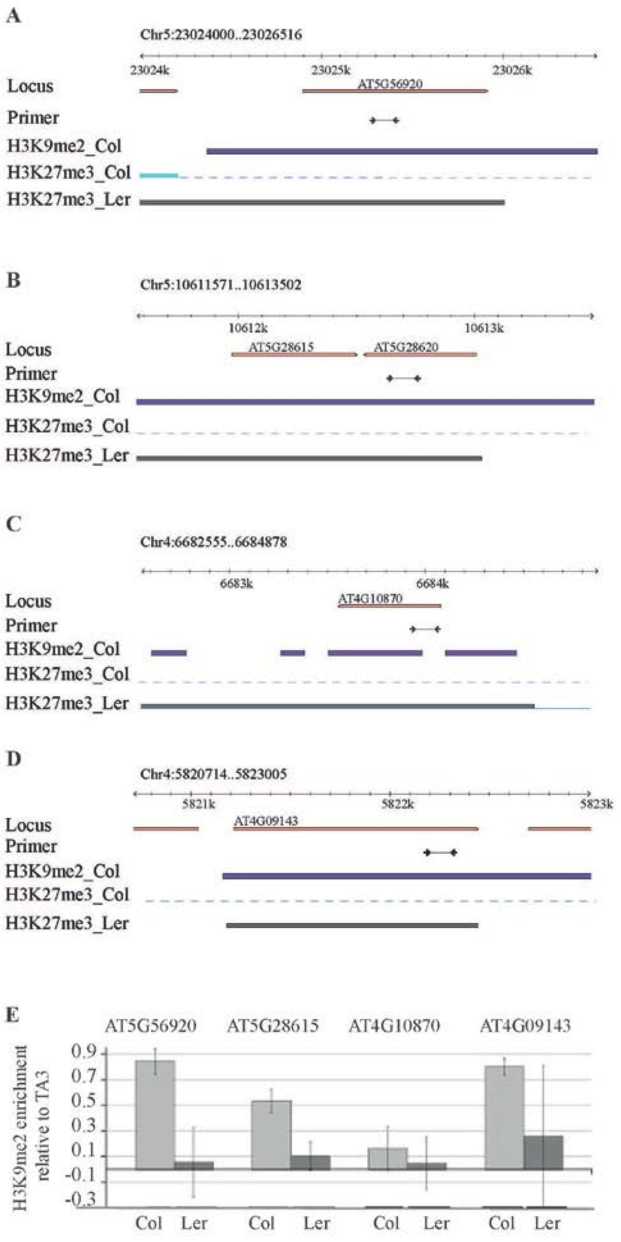

Supplemental Figure 3. **Absence of heterochromatic H3K9me2 from L*er*-specific H3K27me3 targets in L*er* but not Col (A-D)** Schematic representation of four L*er*-specifc H3K27me3 target loci (brown boxes). H3K9me2 enriched regions (purple boxes) are indicated according to Rehrauer et al. [1], H3K27me3-enriched regions in Col (green boxes) are indicated according to the present study. Absence of H3K27me3 in L*er* is symbolized by a dashed line. (**E**) ChIP analysis for H3K9me2 occupancy in Col and L*er* using amplicons as indicated in (A-D). Data are a normalized to the signal detected at the heterochrmatic *TA3* retrotransposon and displayed with the standard error calculated from three biological replicates.

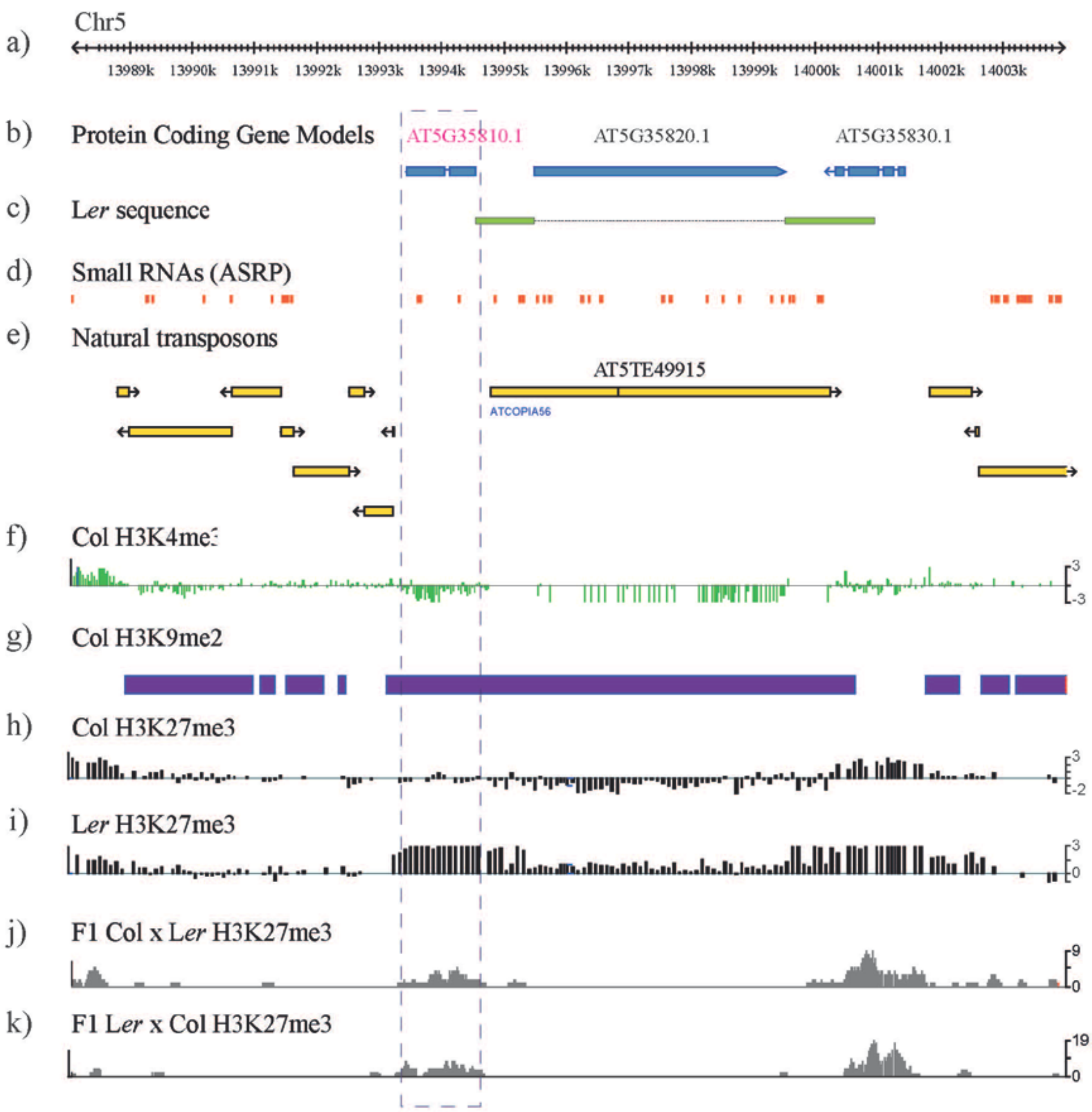

Supplemental Figure 4. A typical example of a L*er*-specific H3K27me3 target that supports the model of heterochromatin spreading. A dashed box indicates a region with differential H3K27me3 between Col and L*er*.Genomic coordinates of a representative region at chromosome 5 from 13988089 to 14003989bp. (**b**) Protein coding gene models are shown as blue bars. (**c**) Sequence comparison of predicted missing region in L*er*. The common sequences between Col and L*er* based on PCR products are shown as green box, regions only present in Col are shown in dashed grey line. (**d**) Locations of small RNAs from ASRP [2]. (**e**) TE annotation based on TAIR TAIR9 genome assembly. (**e**) H3K4me3 signal in this region [3]. (**f**) H3K27me3 signal in Col based on ChIP-chip data. (**g**) H3K9me2 marked regions in purple [4]. (**h**) H3K27me3 signal in Col and (**i**) L*er* based on ChIP-chip data. Both tracks show the scaled mean log2 values (IP/INPUT) for each probe. (**j**) H3K27me3 signal in ColxL*er* and (**k**) L*er* x Col F1 hybrids based on ChIP-Seq data.

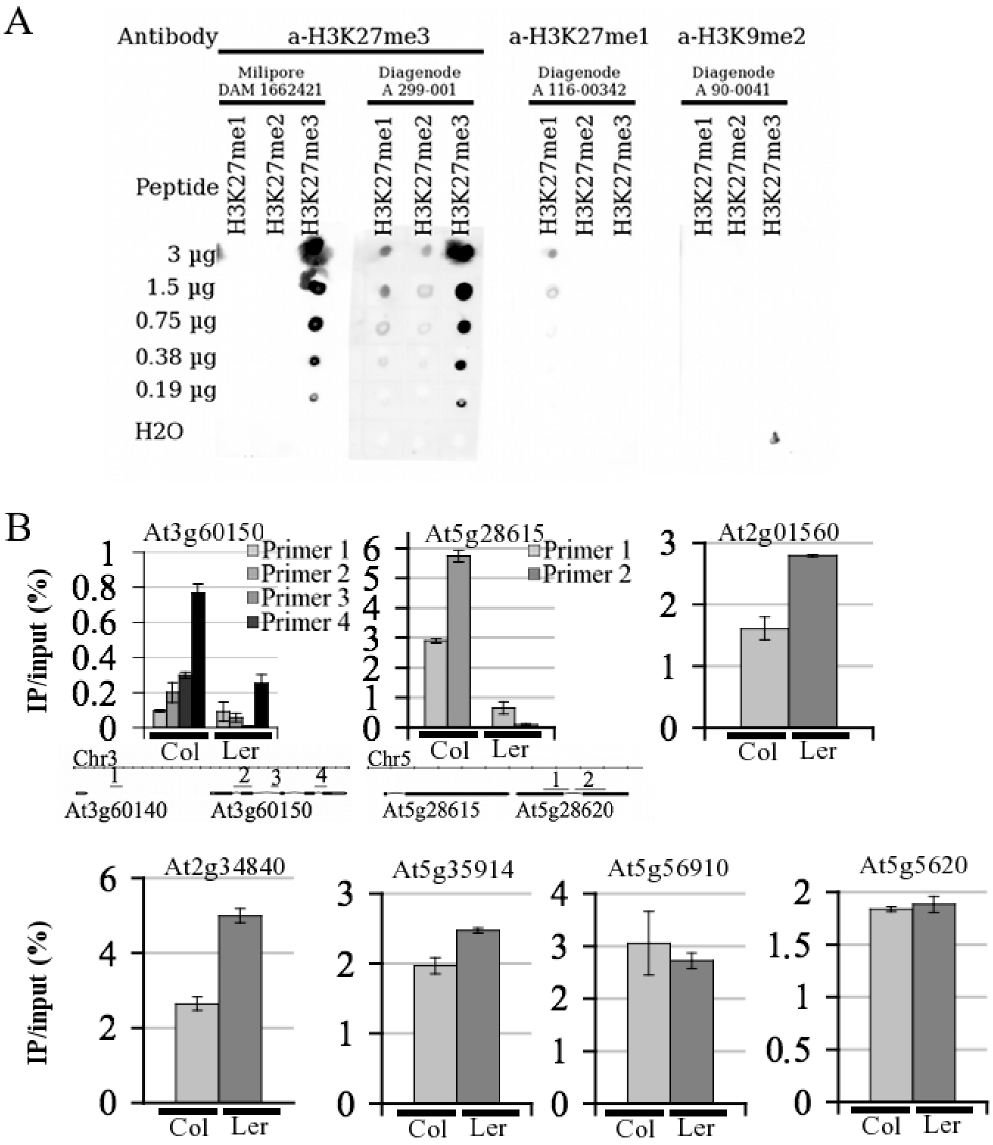

Supplemental Figure 5. **Specificity test of antibodies and H3K27me1 ChIP analysis. (A)** Commercially available antibody preparations were screened in western dot-blots for cross-reactivity against different amount of H3K27me1, H3K27me2 and H3K27me3 peptides. Only antibodies without detectable cross-reactivity were used for further analyses. **(B)** Chromatin from ten day old seedlings was used to perform ChIP for H3K27me1 occupancy. Random sets of confirmed L*er*-specific H3K27me3 targets were chosen to elucidate the enrichment for H3K27me1 using qPCR. Data are shown as ChIP enrichment per input chromatin for Col and L*er* are indicated. Data show the mean of one representative experiment with SE based on three technical replicates.

Supplemental Table 1. H3K27me3 target gene list of Col, L*er*, F1 hybrids Col x L*er* and L*er* x Col.

Supplemental Table 2. Genes that were removed from the analysis after stringent remapping of probes to Col and L*er* scaffolds.

	Gene name	FC:(Ler/Col)	pfp	p-value	experimentally confirmed
L*er*-specific H3K27me3 targets	AT1G21870	3.043	0.1367	0.0006	NA
	AT1G30835	5.0965	0.0075	0	yes
	AT1G35186	3.0756	0.1317	0.0005	NA
	AT1G35400	3.5033	0.0608	0.0001	NA
	AT1G54230	3.032	0.1412	0.0006	NA
	AT1G57565	3.0076	0.1384	0.0007	NA
	AT1G65170	3.382	0.0838	0.0002	NA
	AT1G66300	3.232	0.1042	0.0003	NA
	AT2G01560	3.1715	0.1113	0.0005	yes
	AT2G16830	3.3951	0.0906	0.0002	NA
	AT2G20910	3.9212	0.022	0	yes
	AT2G34840	2.9816	0.1303	0.0006	yes
	AT2G36710	3.0791	0.1332	0.0006	NA
	AT3G46160	3.1138	0.1372	0.0006	NA
	AT3G60150	4.6442	0.0117	0	yes
	AT3G60560	3.4693	0.0861	0.0003	NA
	AT3G60965	2.9695	0.1384	0.0007	NA
	AT4G03566	4.8993	0.01	0	NA
	AT4G09143	3.198	0.1086	0.0004	yes
	AT4G10870	3.214	0.1125	0.0004	NA
	AT4G20480	4.1671	0.0233	0	yes
	AT4G26350	3.9237	0.0264	0	NA
	AT5G02700	3.4585	0.0664	0.0002	NA
	AT5G12910	3.1846	0.1129	0.0004	NA
	AT5G28463	4.8083	0.014	0	yes
	AT5G28610	3.5238	0.076	0.0002	NA
	AT5G28615	3.7386	0.0292	0.0001	yes
	AT5G35810	8.0596	0	0	yes
	AT5G35914	5.206	0.0067	0	no
	AT5G42640	4.2458	0.0225	0	yes
	AT5G56910	3.0358	0.141	0.0007	yes
	AT5G56920	5.9724	0	0	yes
Col-specific H3K27me3 targets	AT5G60610	0.1617	0	0	no
	AT5G28145	0.189	0.005	0	NA
	AT4G24420	0.1837	0.0067	0	yes
	AT1G11450	0.1998	0.0075	0	yes
	AT4G29770	0.1911	0.008	0	no
	AT1G31250	0.2107	0.015	0	no
	AT5G36240	0.2063	0.0157	0	NA
	AT5G19875	0.2584	0.0412	0.0001	no
	AT4G22513	0.2595	0.0656	0.0001	NA
	AT4G32230	0.2525	0.064	0.0001	NA
	AT5G11070	0.2696	0.0791	0.0002	NA

Supplemental Table 3. **L*er*-specifc and Col-specific H3K27me3 targets.** Specific H3K27me3 targets of accessions as indicated (column 1), Arabidopsis gene identifier (column 2), fold-change of median values calculated from all probes over each gene body (column3), percentage of false positives corrected predictions (column 4), p-value (column 5), experimental confirmation (column 6). NA: not assessed.

Gene type	TE_left	Ler specific H3K27me3 target	TE_right	sequence polymorphism (without SNPs)	Confirmed	Target site duplication in Col	Expression in Col	H3K4me3 in Col	H3K9me2 in Col
Protein coding	no	AT1G21870	no	non TE insertion at gene			no	no	no
TE	no	AT1G30835	no	no			yes	yes	no
TE	no	AT1G35186	no	no			no	no	yes
Protein coding	AT1TE42435	AT1G35400	AT1TE42440	no			no	no	yes
Protein coding	no	AT1G54230	no	no			no	no	no
Protein coding	AT1TE70420	AT1G57565	no	no			no	no	yes
Protein coding	no	AT1G65170	no	non TE insertion at gene in Col			no	no	no
Protein coding	no	AT1G66300	AT1TE81190	flanking TE insertion in Col			no	no	no
Protein coding	AT2TE01000	AT2G01560	AT2TE01010	flanking TE insertion in Col			no	no	no
Pseudogene	AT2TE29725	AT2G16830	AT2TE29730	flanking TE insertion in Col			no	no	yes
Pseudogene	AT2TE37940	AT2G20910	no	non TE insertion at gene in Col			no	no	yes
Protein coding	AT2TE65225	AT2G34840	AT2TE65230	TE insertion in gene in Col	yes	yes	yes	no	yes
Protein coding	AT2TE68590	AT2G36710	no	rearrangement			no	no	yes
Protein coding	no	AT3G46160	no	no			no	no	no
Protein coding	no	AT3G60150	no	insertion at gene in Ler			yes	yes	yes
Protein coding	no	AT3G60560	AT3TE91170	flanking TE insertion in Col			no	no	no
TE	AT3TE91830	AT3G60965	no	insertion at gene in Col			no	no	no
Protein coding	no	AT4G03566	no	no			no	no	yes
Pseudogene	no	AT4G09143	AT4TE24480	flanking TE insertion in Col			no	no	yes
Protein coding	AT4TE28665	AT4G10870	AT4TE28670	flanking TE insertion in Col			no	no	yes
Protein coding	AT4TE50620	AT4G20480	AT4TE50630	flanking TE insertion in Col			yes	yes	no
Protein coding	no	AT4G26350	AT4TE62660	NA			no	no	yes
Protein coding	no	AT5G02700	AT5TE02190	rearrangement			no	no	yes
Protein coding	AT5TE14765	AT5G12910	no	no			no	no	no
Protein coding	AT5TE37885	AT5G28463	AT5TE37900	no			no	no	yes
Protein coding	AT5TE38680	AT5G28610	no	no			no	no	yes
Protein coding	no	AT5G28615	no	no			no	no	yes
Protein coding	AT5TE49910	AT5G35810	AT5TE49915	flanking TE insertion in Col	yes	yes	no	no	yes
TE	AT5TE50065	AT5G35914	no	no			no	no	no
Protein coding	AT5TE61720	AT5G42640	AT5TE61725	flanking TE insertion in Col	yes	yes	no	no	yes
Protein coding	no	AT5G56910	AT5TE82820	flanking TE insertion in Col	yes	no	yes	yes	no
Protein coding	AT5TE82820	AT5G56920	AT5TE82825	flanking TE insertion in Col	yes	yes	no	no	yes

Supplemental Table 4. Genomic features associated with L*er*-specific H3K27me3 targets

AGI	SEQ FW	SEQ REV	PRODUCT SIZE
Col-specific H3K27me3			
AT1G11450	TTCAAAATGGGCACAAAACA	CGTCTCCGTCTGGTCGTATAG	218
AT1G31250	TTCCCTAGCCTCGATTCGTA	GCTAACCATCAAGCATTCCAA	473
AT4G24420	AGGACCCAACGAGTGATGAA	ACTTGTTGTCAAGGCCAACC	165
AT4G29770	AAGCTGGGTATGCAACAACC	TTGATCTTCTCCGCCTCAAC	245
AT5G19875	ACACCCTGGACCACCTCAAGC	GCTGCTACGCCCCAAGGGGA	154
AT5G60610	AACGCCTTGTTGAAGTTTGG	AAATCTGCGATTGGAATTGG	226
Ler-specific H3K27me3			
AT3G60150			
PRIMER 1	CGGCAAGGCGATGTATATTA	TGGTGACGTGATGAAAGAGA	198
PRIMER 2/expression analysis	CAGCTCCGATTTCAGGAGTA	AAGGGAGAAGGTGGTACTGG	255
PRIMER 3	AGTGTGGCCTCTCTTGTACG	AAGATTGACAAGCTGCATCC	146
PRIMER 4	TCGGCATGAAGCTAGAAACT	ATAAGGAAGCAATGCAGCAG	187
AT5G28615			
expression analysis	CCCCAATTCACTGGTTTGTC	TCTCCGGGTTTGGTAGAATG	133
PRIMER 1	CCACCCAGTTTATGGCTTGT	GCGGAATGAAGACGAATGTT	136
PRIMER 2	CCGCCAATTATAGGTGTGCT	GAGCCACAAAGGTGGGTTTA	159
AT1G30835	AATGGTTAGTGGTCCTCAAA	AATCATCATTGCCACGAGGT	175
AT1G35400	TGAGGTCTGCCAAGGAGTTT	ATCACTTCCGGTGTTGCTTC	109
AT2G01560	TCTCCACAGGAATTGGGGTA	AGCGTTCAAAGGGGAAAAAT	230
AT2G20910	GAGCAGCGATTTCATTGTGA	TAGCCTCCTCTTGACGCATT	215
AT2G34840	CAACTTGCTAGCGCTTGGTT	CAAACAGCTTTGCCATTCAA	217
AT4G09143	GAAACCTTGACCCCGACTCT	TTGCATCGTGAACCTTCCTT	160
AT4G10870	TCTATAAATCTTCGTATATGGACC AC	GGCTCCATTGTTACATAGGGT TT	151
AT4G20480	TATCAAGTCCCGGCCAGTAA	TGAGGAGGAGAAGTGCAGTG	103
AT4G24420	AAT GGC AAC GTC CTC TGT TC	CAG CTG TAG GGG GAT TGG TA	173
AT5G28463	GCAGTTCTTGGTGGAAGAGC	CCTCCGGAAACAAATCAAGA	197
AT5G35810	GCTGTGAAGGCAAGAACCAT	AT4G03443AT5G16430	168
AT5G35914	ATGCGACGACAAATCACAAA	CTTGAATTTTGGCCCACTGT	215
AT5G42640	TGGTTCGATCCATGTTCATT	ATCAGCACGGGAAACAGAAT	179
AT5G56910	ACGAACCAATGCTGATGTGA	GCTGAGACATGCTCGACAAA	102
AT5G56920	TCTCGATTAAATGGGAGCAA	GGTTCCATGAGCTTCGTTGT	152
FT	TGCGTATTTGAGTTCGGACA	AGAACAAGGGATCCTTCAGGT	165
PP2A/AT1G13320	CAGCAACGAATTGTGTTTGG	AAATACGCCCAACGAACAAA	155
TE-analysis			
AT2G34840	AAGTCATGCATCCCTCGTTC	CAACCAAGCGCTAGCAAGTT	2839 in Col
AT5G35810	CAGATTGTGCAGCATCAAAGA	GAGCCAAAGGTGTCCAACTC	6568 in Col
AT5G42640	CAGATTTTTGGACCCACCAT	GAGATTTTCCGGCGATATGA	7889 in Col
AT5G56910	ACGAACCAATGCTGATGTGA	GGTTCCATGAGCTTCGTTGT	2071 in Col
AT5G56920	TCTCGATTAAATGGGAGCAA	GCGATCCAAGCAAGAGTTTC	3286 in Col

Supplemental Table 5. Oligonucleotide sequences used in this study.

Supplemental Methods

Dot blot

The specificity of commercially available antibodies was assessed by western dot-blots. H3K27me1, H3K7me2 and H3K27me3 peptides were spotted with different concentrations on an activated PVDF membrane. The membrane was blocked with 5% milk in TBST and incubated for 1 ½ hour with the antibody of interest diluted 1:10.000 in TBST. After washing three times with TBST for 15 min each, the membrane was incubated with an antibody against rabbit IgG coupled with a horse raddish peroxidase (HRP). Detection was performed using Pierce ECL Western Blotting Substrate (Pierce; No. 32106) and SuperSignal West Dura Chemiluminescent Substrate (Pierce; No. 37071).

References

1. Rehrauer H, Aquino C, Gruissem W, Henz SR, Hilson P, Laubinger S, Naouar N, Patrignani A, Rombauts S, Shu H *et al*: AGRONOMICS1: a new resource for Arabidopsis transcriptome profiling. *Plant Physiol* 2010, 152(2):487-499.
2. Backman TW, Sullivan CM, Cumbie JS, Miller ZA, Chapman EJ, Fahlgren N, Givan SA, Carrington JC, Kasschau KD: Update of ASRP: the Arabidopsis Small RNA Project database. *Nucleic Acids Res* 2008, 36(Database issue):D982-985.
3. Oh S, Park S, van Nocker S: Genic and global functions for Paf1C in chromatin modification and gene expression in Arabidopsis. *PLoS Genet* 2008, 4(8):e1000077.
4. Bernatavichute YV, Zhang X, Cokus S, Pellegrini M, Jacobsen SE: Genome-wide association of histone H3 lysine nine methylation with CHG DNA methylation in Arabidopsis thaliana. *PLoS One* 2008, 3(9):e3156.
